# Supplementary material for: De novo oviduct transcriptome of the moor frog Rana arvalis: a quest for maternal effect candidate genes
Source: PeerJ. 2018 Aug 16;6:e5452. doi: 10.7717/peerj.5452 (PMC6098945; doi:10.7717/peerj.5452)
Supplement: Supplemental Information 1 [file peerj-06-5452-s001.docx]

**Supplementary Information**

**De novo oviduct transcriptome of the moor frog *Rana arvalis*: a quest for maternal effect candidate genes**

Longfei Shu*^1^, Jie Qiu^2^ and Katja Räsänen^1^

**Affiliations:**

*^1^Eawag, Department of Aquatic Ecology, Switzerland and ETH Zürich, Institute of Integrative Biology, Switzerland*

^2^*Institutue of Crop Science & Institute of Bioinformatics, College of Agriculture and Biotechnology, Zhejiang University, Hangzhou 310058, China*

**Corresponding author:**

Dr. Longfei Shu

Eawag, Department of Aquatic Ecology, Switzerland and ETH Zürich, Institute of Integrative Biology, CH-8600 Dübendorf, Switzerland;

Email: [longfei.shu@wustl.edu](mailto:longfei.shu@wustl.edu); Phone: +1-314-445-8037.

***Current address:** Department of Biology, Washington University in St. Louis, St. Louis, MO 63130, USA

**List of supplementary materials**

**Figure S1. Statistics of SSR classification from *R. arvalis* transcriptome based on oviduct samples from seven individuals**

**Figure S2. Statistics of SNP numbers in seven cDNA libraries**

**Figure S3. Differential expression analysis among three populations**

**Table S1. Distribution of SSRs in the *R. arvalis* oviduct transcriptome**

**Table S2. KEGG annotation of *R. arvalis* oviduct transcriptome**

**Tables S3-S5. Enrichment of KEGG pathway in the differentially expressed unigenes between population pairs**

**SI2. List of unigenes that are significantly correlated with embryonic acid tolerance**

**SI3. List of unigenes with SNP differentiation between the S and T population**


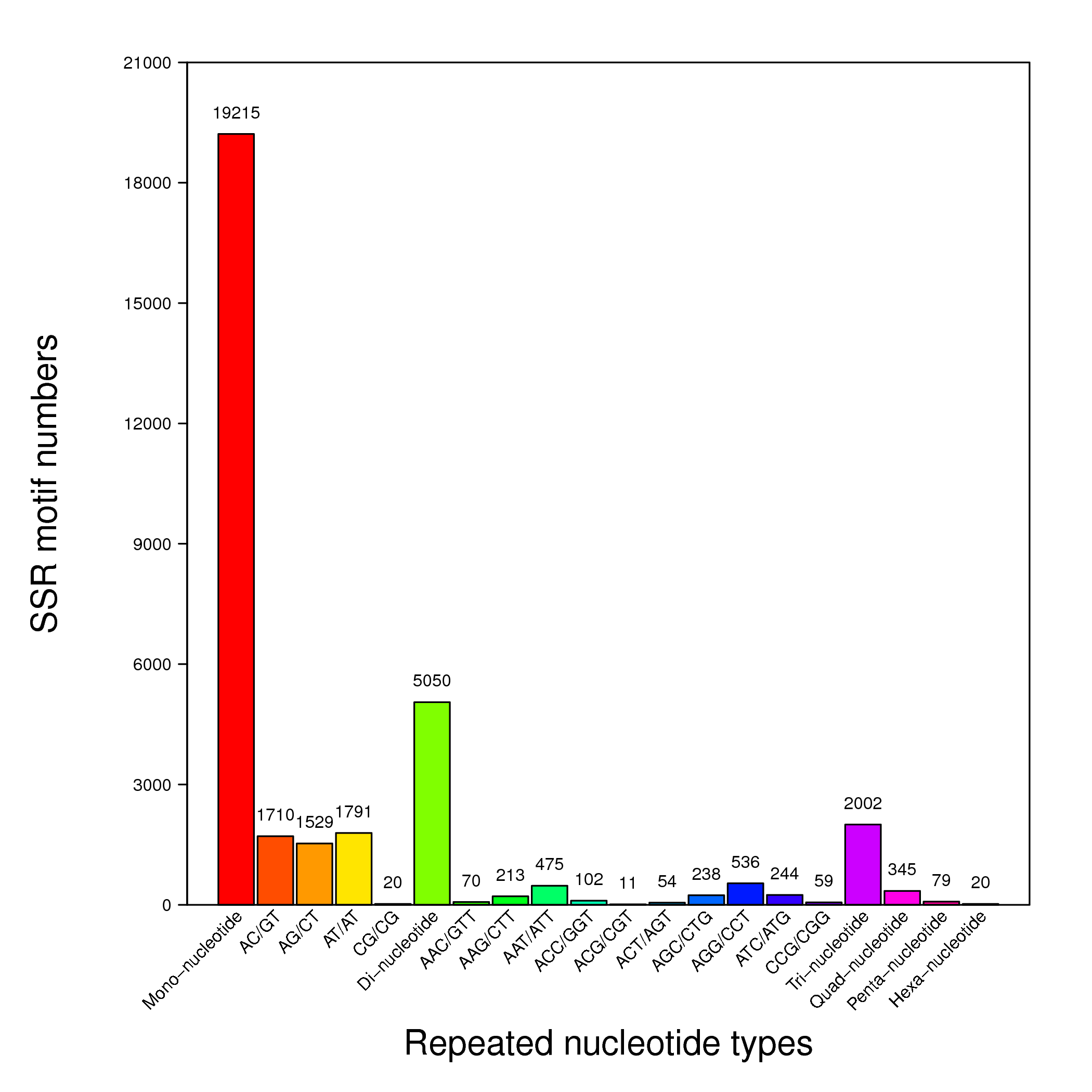


**Figure S1 Statistics of SSR classification from *R. arvalis* transcriptome based on oviduct samples from seven individuals.** Among Mono-nucleotide repeats, (A/T)n was the most abundant motif. Among Di-nucleotide repeats, the most abundant repeats were (AC/GT)n, (AG/CT)n and (AT/AT)n. Among Tri-nucleotide motifs, the most abundant repeats were (AAT/ATT)n and (AGG/CCT)n. Among Quad-nucleotide motifs, the most abundant repeats were (AGAT/ATCT)n and (AAAG/CTTT)n. Among Penta-nucleotide motifs, the most abundant repeats were (AGAGG/CCTCT)n and (AAAAT/ATTTT)n. Among Hexa-nucleotide motifs, the most abundant repeats were (AAAAAT/ATTTTT)n and (AGGGGC/CCCCTG)n. After filtration, 8 073 high-quality primer pairs amplifying a single product were obtained.


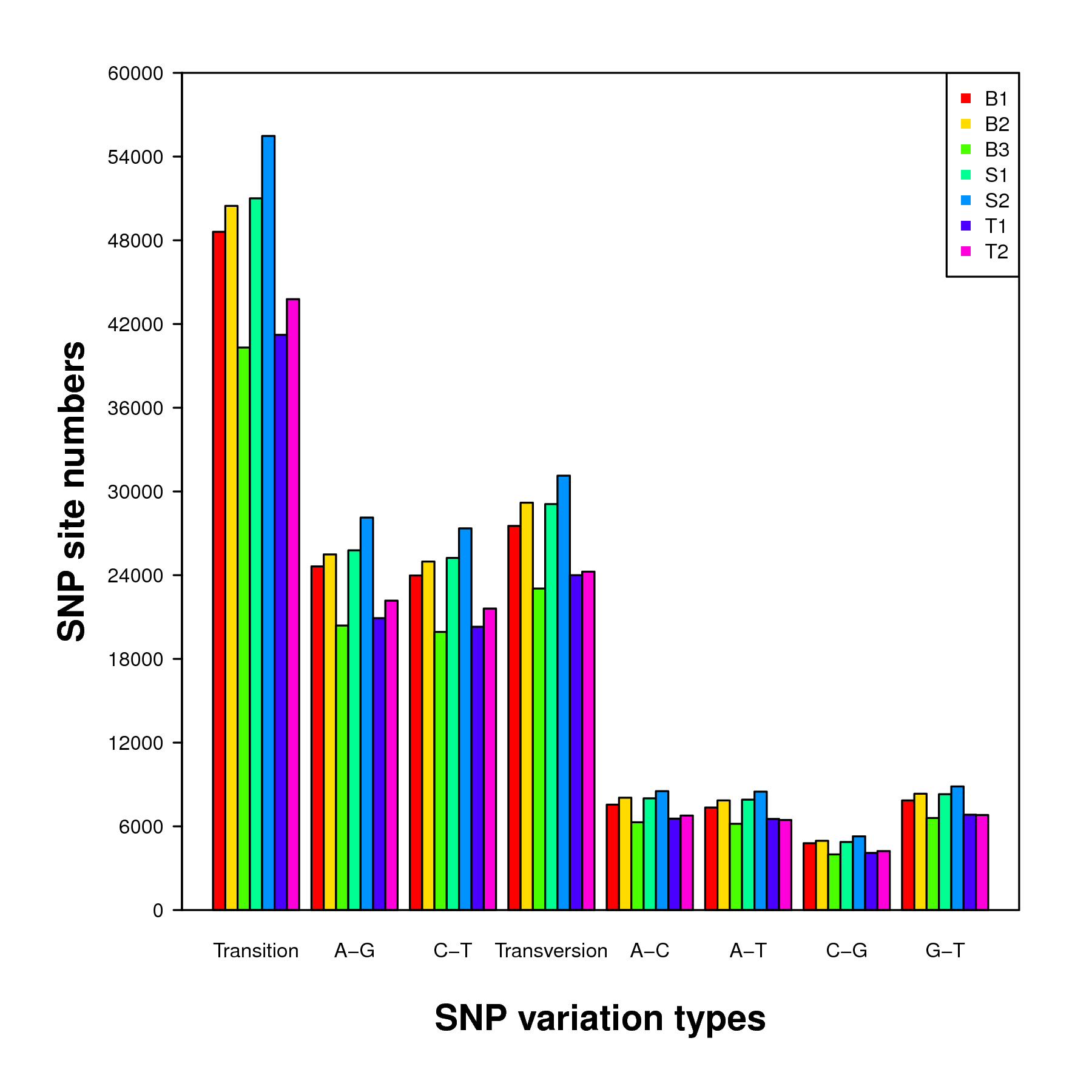


**Figure S2 Statistics of SNP numbers in seven cDNA libraries.** The X-axis indicates the SNP types, the Y-axis the number of SNPs for a given type. The colors indicate individual females from different populations (acid origin population, T, neutral origin population, S, and intermediate pH population, B). The numbers 1 to 3 indicate the individual identity of the females.

**
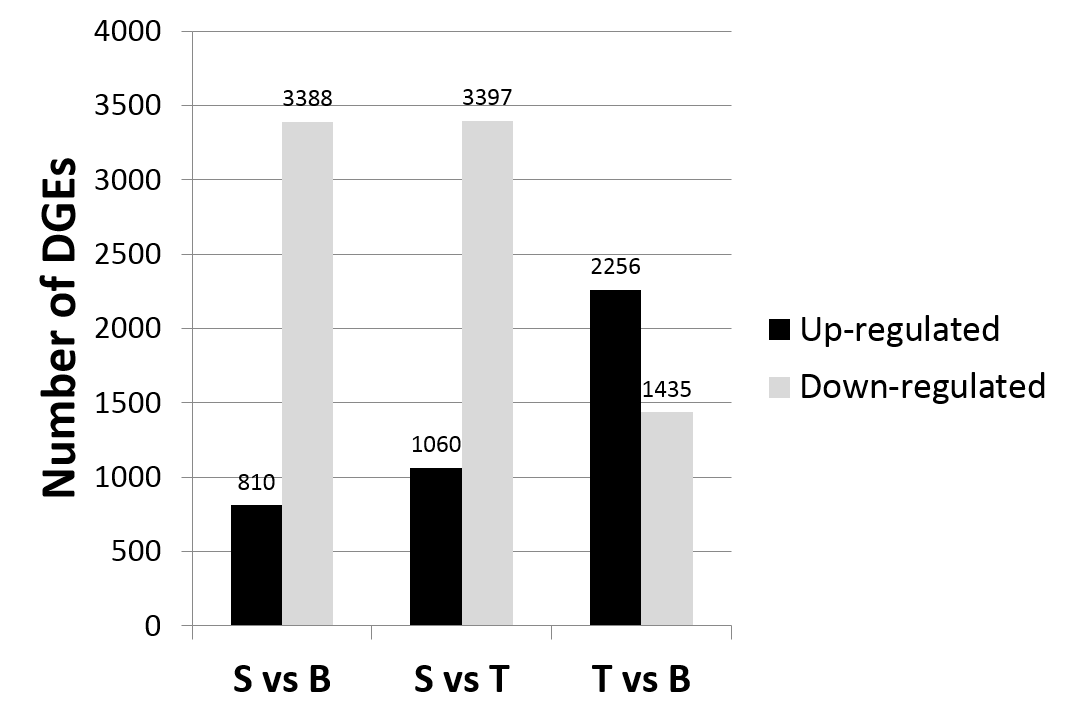
**

**Figure S3 Differential expression analysis among three populations.** The X-axis indicates group comparisons, the Y-axis the number of DGEs.

**Table S1. Distribution of SSRs in the *R. arvalis* oviduct transcriptome based on seven individuals.**

| **Number of repeats** | **Mono-nucleotide repeats** | **Di-nucleotide repeats** | **Tri-nucleotide repeats** | **Quad-nucleotide repeat** | **Penta-nucleotide repeats** | **Hexa-nucleotide repeats** |
| --- | --- | --- | --- | --- | --- | --- |
| 4 | 0 | 0 | 0 | 0 | 69 | 20 |
| 5 | 0 | 0 | 1,180 | 292 | 10 | 0 |
| 6 | 0 | 2,048 | 472 | 50 | 0 | 0 |
| 7 | 0 | 1,068 | 286 | 1 | 0 | 0 |
| 8 | 0 | 645 | 60 | 1 | 0 | 0 |
| 9 | 0 | 467 | 3 | 1 | 0 | 0 |
| 10 | 0 | 456 | 1 | 0 | 0 | 0 |
| 11 | 0 | 348 | 0 | 0 | 0 | 0 |
| 12 | 5,141 | 16 | 0 | 0 | 0 | 0 |
| 13 | 3,858 | 1 | 0 | 0 | 0 | 0 |
| 14 | 2,742 | 0 | 0 | 0 | 0 | 0 |
| 15 | 2,105 | 0 | 0 | 0 | 0 | 0 |
| 16 | 1,488 | 0 | 0 | 0 | 0 | 0 |
| 17 | 977 | 0 | 0 | 0 | 0 | 0 |
| 18 | 826 | 0 | 0 | 0 | 0 | 0 |
| 19 | 750 | 0 | 0 | 0 | 0 | 0 |
| 20 | 702 | 0 | 0 | 0 | 0 | 0 |
| 21 | 397 | 1 | 0 | 0 | 0 | 0 |
| 22 | 172 | 0 | 0 | 0 | 0 | 0 |
| 23 | 53 | 0 | 0 | 0 | 0 | 0 |
| 24 | 4 | 0 | 0 | 0 | 0 | 0 |
| Sub Total | 19,215 | 5,050 | 2,002 | 345 | 79 | 20 |

**Table S2. KEGG annonation of *R. arvalis* oviduct transcriptome.**

| **Pathway** | **All genes with pathway annotation (31405)** | **Pathway ID** | **Level 1** |
| --- | --- | --- | --- |
| Metabolic pathways | 3796 (12.09%) | ko01100 | Metabolism |
| Purine metabolism | 1910 (6.08%) | ko00230 | Metabolism |
| Huntington's disease | 1612 (5.13%) | ko05016 | Human Diseases |
| Pathways in cancer | 1405 (4.47%) | ko05200 | Human Diseases |
| Regulation of actin cytoskeleton | 1202 (3.83%) | ko04810 | Cellular Processes |
| Focal adhesion | 1161 (3.7%) | ko04510 | Cellular Processes |
| Calcium signaling pathway | 1159 (3.69%) | ko04020 | Environmental Information Processing |
| Influenza A | 1121 (3.57%) | ko05164 | Human Diseases |
| Lysine degradation | 1120 (3.57%) | ko00310 | Metabolism |
| Ubiquitin mediated proteolysis | 1097 (3.49%) | ko04120 | Genetic Information Processing |
| Morphine addiction | 1081 (3.44%) | ko05032 | Human Diseases |
| Herpes simplex infection | 1077 (3.43%) | ko05168 | Human Diseases |
| Epstein-Barr virus infection | 1058 (3.37%) | ko05169 | Human Diseases |
| Fc gamma R-mediated phagocytosis | 1051 (3.35%) | ko04666 | Organismal Systems |
| T cell receptor signaling pathway | 967 (3.08%) | ko04660 | Organismal Systems |
| Protein processing in endoplasmic reticulum | 950 (3.02%) | ko04141 | Genetic Information Processing |
| Spliceosome | 910 (2.9%) | ko03040 | Genetic Information Processing |
| Cell adhesion molecules (CAMs) | 886 (2.82%) | ko04514 | Environmental Information Processing |
| Olfactory transduction | 886 (2.82%) | ko04740 | Organismal Systems |
| Tight junction | 859 (2.74%) | ko04530 | Cellular Processes |
| Endocytosis | 852 (2.71%) | ko04144 | Cellular Processes |
| MAPK signaling pathway | 797 (2.54%) | ko04010 | Environmental Information Processing |
| RNA transport | 792 (2.52%) | ko03013 | Genetic Information Processing |
| Taste transduction | 790 (2.52%) | ko04742 | Organismal Systems |
| Pyrimidine metabolism | 773 (2.46%) | ko00240 | Metabolism |
| Vascular smooth muscle contraction | 763 (2.43%) | ko04270 | Organismal Systems |
| Alzheimer's disease | 753 (2.4%) | ko05010 | Human Diseases |
| Primary immunodeficiency | 729 (2.32%) | ko05340 | Human Diseases |
| HTLV-I infection | 683 (2.17%) | ko05166 | Human Diseases |
| Hepatitis C | 646 (2.06%) | ko05160 | Human Diseases |
| Amoebiasis | 639 (2.03%) | ko05146 | Human Diseases |
| Ribosome biogenesis in eukaryotes | 636 (2.03%) | ko03008 | Genetic Information Processing |
| Insulin signaling pathway | 634 (2.02%) | ko04910 | Organismal Systems |
| Measles | 629 (2%) | ko05162 | Human Diseases |
| Transcriptional misregulation in cancer | 615 (1.96%) | ko05202 | Human Diseases |
| Salmonella infection | 614 (1.96%) | ko05132 | Human Diseases |
| Phagosome | 575 (1.83%) | ko04145 | Cellular Processes |
| Chemokine signaling pathway | 571 (1.82%) | ko04062 | Organismal Systems |
| NF-kappa B signaling pathway | 570 (1.81%) | ko04064 | Environmental Information Processing |
| Dilated cardiomyopathy | 566 (1.8%) | ko05414 | Human Diseases |
| Tuberculosis | 563 (1.79%) | ko05152 | Human Diseases |
| Adherens junction | 561 (1.79%) | ko04520 | Cellular Processes |
| RNA polymerase | 546 (1.74%) | ko03020 | Genetic Information Processing |
| Wnt signaling pathway | 544 (1.73%) | ko04310 | Environmental Information Processing |
| Hypertrophic cardiomyopathy (HCM) | 528 (1.68%) | ko05410 | Human Diseases |
| Bile secretion | 498 (1.59%) | ko04976 | Organismal Systems |
| Lysosome | 493 (1.57%) | ko04142 | Cellular Processes |
| ECM-receptor interaction | 484 (1.54%) | ko04512 | Environmental Information Processing |
| Neurotrophin signaling pathway | 476 (1.52%) | ko04722 | Organismal Systems |
| Viral myocarditis | 475 (1.51%) | ko05416 | Human Diseases |
| mRNA surveillance pathway | 461 (1.47%) | ko03015 | Genetic Information Processing |
| Pathogenic Escherichia coli infection | 458 (1.46%) | ko05130 | Human Diseases |
| Axon guidance | 456 (1.45%) | ko04360 | Organismal Systems |
| Osteoclast differentiation | 440 (1.4%) | ko04380 | Organismal Systems |
| Leukocyte transendothelial migration | 438 (1.39%) | ko04670 | Organismal Systems |
| Vibrio cholerae infection | 438 (1.39%) | ko05110 | Human Diseases |
| Hedgehog signaling pathway | 434 (1.38%) | ko04340 | Environmental Information Processing |
| Basal cell carcinoma | 430 (1.37%) | ko05217 | Human Diseases |
| B cell receptor signaling pathway | 429 (1.37%) | ko04662 | Organismal Systems |
| ABC transporters | 425 (1.35%) | ko02010 | Environmental Information Processing |
| Prostate cancer | 414 (1.32%) | ko05215 | Human Diseases |
| Steroid hormone biosynthesis | 412 (1.31%) | ko00140 | Metabolism |
| Bacterial invasion of epithelial cells | 411 (1.31%) | ko05100 | Human Diseases |
| RIG-I-like receptor signaling pathway | 405 (1.29%) | ko04622 | Organismal Systems |
| Cardiac muscle contraction | 400 (1.27%) | ko04260 | Organismal Systems |
| Toxoplasmosis | 391 (1.25%) | ko05145 | Human Diseases |
| Dopaminergic synapse | 389 (1.24%) | ko04728 | Organismal Systems |
| Cell cycle | 388 (1.24%) | ko04110 | Cellular Processes |
| Oocyte meiosis | 381 (1.21%) | ko04114 | Cellular Processes |
| Alcoholism | 380 (1.21%) | ko05034 | Human Diseases |
| Primary bile acid biosynthesis | 368 (1.17%) | ko00120 | Metabolism |
| Shigellosis | 362 (1.15%) | ko05131 | Human Diseases |
| Small cell lung cancer | 359 (1.14%) | ko05222 | Human Diseases |
| Natural killer cell mediated cytotoxicity | 354 (1.13%) | ko04650 | Organismal Systems |
| Long-term potentiation | 353 (1.12%) | ko04720 | Organismal Systems |
| Basal transcription factors | 352 (1.12%) | ko03022 | Genetic Information Processing |
| Neuroactive ligand-receptor interaction | 350 (1.11%) | ko04080 | Environmental Information Processing |
| Progesterone-mediated oocyte maturation | 350 (1.11%) | ko04914 | Organismal Systems |
| Serotonergic synapse | 348 (1.11%) | ko04726 | Organismal Systems |
| ErbB signaling pathway | 329 (1.05%) | ko04012 | Environmental Information Processing |
| Protein digestion and absorption | 327 (1.04%) | ko04974 | Organismal Systems |
| Salivary secretion | 327 (1.04%) | ko04970 | Organismal Systems |
| Gastric acid secretion | 315 (1%) | ko04971 | Organismal Systems |
| Oxidative phosphorylation | 311 (0.99%) | ko00190 | Metabolism |
| Parkinson's disease | 310 (0.99%) | ko05012 | Human Diseases |
| Vasopressin-regulated water reabsorption | 310 (0.99%) | ko04962 | Organismal Systems |
| Melanogenesis | 308 (0.98%) | ko04916 | Organismal Systems |
| Chronic myeloid leukemia | 308 (0.98%) | ko05220 | Human Diseases |
| Amyotrophic lateral sclerosis (ALS) | 305 (0.97%) | ko05014 | Human Diseases |
| Starch and sucrose metabolism | 304 (0.97%) | ko00500 | Metabolism |
| Renal cell carcinoma | 302 (0.96%) | ko05211 | Human Diseases |
| GnRH signaling pathway | 299 (0.95%) | ko04912 | Organismal Systems |
| Phosphatidylinositol signaling system | 298 (0.95%) | ko04070 | Environmental Information Processing |
| TGF-beta signaling pathway | 292 (0.93%) | ko04350 | Environmental Information Processing |
| NOD-like receptor signaling pathway | 291 (0.93%) | ko04621 | Organismal Systems |
| Hematopoietic cell lineage | 288 (0.92%) | ko04640 | Organismal Systems |
| RNA degradation | 286 (0.91%) | ko03018 | Genetic Information Processing |
| mTOR signaling pathway | 285 (0.91%) | ko04150 | Environmental Information Processing |
| Endometrial cancer | 285 (0.91%) | ko05213 | Human Diseases |
| Gap junction | 284 (0.9%) | ko04540 | Cellular Processes |
| Amino sugar and nucleotide sugar metabolism | 283 (0.9%) | ko00520 | Metabolism |
| Maturity onset diabetes of the young | 281 (0.89%) | ko04950 | Human Diseases |
| Chagas disease (American trypanosomiasis) | 280 (0.89%) | ko05142 | Human Diseases |
| Jak-STAT signaling pathway | 275 (0.88%) | ko04630 | Environmental Information Processing |
| Pancreatic cancer | 271 (0.86%) | ko05212 | Human Diseases |
| Glioma | 269 (0.86%) | ko05214 | Human Diseases |
| Pancreatic secretion | 260 (0.83%) | ko04972 | Organismal Systems |
| Apoptosis | 260 (0.83%) | ko04210 | Cellular Processes |
| Colorectal cancer | 258 (0.82%) | ko05210 | Human Diseases |
| Acute myeloid leukemia | 255 (0.81%) | ko05221 | Human Diseases |
| Arrhythmogenic right ventricular cardiomyopathy (ARVC) | 255 (0.81%) | ko05412 | Human Diseases |
| Ribosome | 254 (0.81%) | ko03010 | Genetic Information Processing |
| Pertussis | 244 (0.78%) | ko05133 | Human Diseases |
| Fc epsilon RI signaling pathway | 244 (0.78%) | ko04664 | Organismal Systems |
| Notch signaling pathway | 243 (0.77%) | ko04330 | Environmental Information Processing |
| Peroxisome | 241 (0.77%) | ko04146 | Cellular Processes |
| VEGF signaling pathway | 240 (0.76%) | ko04370 | Environmental Information Processing |
| Cholinergic synapse | 236 (0.75%) | ko04725 | Organismal Systems |
| Cytokine-cytokine receptor interaction | 236 (0.75%) | ko04060 | Environmental Information Processing |
| Non-small cell lung cancer | 235 (0.75%) | ko05223 | Human Diseases |
| Long-term depression | 232 (0.74%) | ko04730 | Organismal Systems |
| Glutamatergic synapse | 231 (0.74%) | ko04724 | Organismal Systems |
| PPAR signaling pathway | 230 (0.73%) | ko03320 | Organismal Systems |
| Glycolysis / Gluconeogenesis | 229 (0.73%) | ko00010 | Metabolism |
| Inositol phosphate metabolism | 224 (0.71%) | ko00562 | Metabolism |
| Adipocytokine signaling pathway | 224 (0.71%) | ko04920 | Organismal Systems |
| Glycerophospholipid metabolism | 216 (0.69%) | ko00564 | Metabolism |
| Complement and coagulation cascades | 214 (0.68%) | ko04610 | Organismal Systems |
| Carbohydrate digestion and absorption | 207 (0.66%) | ko04973 | Organismal Systems |
| Synaptic vesicle cycle | 205 (0.65%) | ko04721 | Organismal Systems |
| Melanoma | 204 (0.65%) | ko05218 | Human Diseases |
| Legionellosis | 203 (0.65%) | ko05134 | Human Diseases |
| Toll-like receptor signaling pathway | 202 (0.64%) | ko04620 | Organismal Systems |
| Amphetamine addiction | 201 (0.64%) | ko05031 | Human Diseases |
| Antigen processing and presentation | 194 (0.62%) | ko04612 | Organismal Systems |
| Epithelial cell signaling in Helicobacter pylori infection | 187 (0.6%) | ko05120 | Human Diseases |
| Thyroid cancer | 182 (0.58%) | ko05216 | Human Diseases |
| Metabolism of xenobiotics by cytochrome P450 | 181 (0.58%) | ko00980 | Metabolism |
| p53 signaling pathway | 179 (0.57%) | ko04115 | Cellular Processes |
| Fanconi anemia pathway | 179 (0.57%) | ko03460 | Genetic Information Processing |
| Valine, leucine and isoleucine degradation | 177 (0.56%) | ko00280 | Metabolism |
| Retinol metabolism | 172 (0.55%) | ko00830 | Metabolism |
| Pentose and glucuronate interconversions | 170 (0.54%) | ko00040 | Metabolism |
| Retrograde endocannabinoid signaling | 168 (0.53%) | ko04723 | Organismal Systems |
| Arachidonic acid metabolism | 166 (0.53%) | ko00590 | Metabolism |
| Staphylococcus aureus infection | 165 (0.53%) | ko05150 | Human Diseases |
| Galactose metabolism | 163 (0.52%) | ko00052 | Metabolism |
| N-Glycan biosynthesis | 163 (0.52%) | ko00510 | Metabolism |
| Dorso-ventral axis formation | 162 (0.52%) | ko04320 | Organismal Systems |
| Prion diseases | 162 (0.52%) | ko05020 | Human Diseases |
| Drug metabolism - cytochrome P450 | 161 (0.51%) | ko00982 | Metabolism |
| Bladder cancer | 161 (0.51%) | ko05219 | Human Diseases |
| Arginine and proline metabolism | 160 (0.51%) | ko00330 | Metabolism |
| Type II diabetes mellitus | 159 (0.51%) | ko04930 | Human Diseases |
| Fatty acid metabolism | 158 (0.5%) | ko00071 | Metabolism |
| Leishmaniasis | 158 (0.5%) | ko05140 | Human Diseases |
| GABAergic synapse | 157 (0.5%) | ko04727 | Organismal Systems |
| Endocrine and other factor-regulated calcium reabsorption | 154 (0.49%) | ko04961 | Organismal Systems |
| Rheumatoid arthritis | 154 (0.49%) | ko05323 | Human Diseases |
| Aminoacyl-tRNA biosynthesis | 154 (0.49%) | ko00970 | Genetic Information Processing |
| Cytosolic DNA-sensing pathway | 152 (0.48%) | ko04623 | Organismal Systems |
| Nucleotide excision repair | 151 (0.48%) | ko03420 | Genetic Information Processing |
| Pyruvate metabolism | 149 (0.47%) | ko00620 | Metabolism |
| Systemic lupus erythematosus | 143 (0.46%) | ko05322 | Human Diseases |
| Fructose and mannose metabolism | 139 (0.44%) | ko00051 | Metabolism |
| Mineral absorption | 138 (0.44%) | ko04978 | Organismal Systems |
| Drug metabolism - other enzymes | 138 (0.44%) | ko00983 | Metabolism |
| Citrate cycle (TCA cycle) | 134 (0.43%) | ko00020 | Metabolism |
| Propanoate metabolism | 132 (0.42%) | ko00640 | Metabolism |
| Cysteine and methionine metabolism | 130 (0.41%) | ko00270 | Metabolism |
| Ascorbate and aldarate metabolism | 130 (0.41%) | ko00053 | Metabolism |
| Aldosterone-regulated sodium reabsorption | 127 (0.4%) | ko04960 | Organismal Systems |
| Phototransduction - fly | 125 (0.4%) | ko04745 | Organismal Systems |
| Phototransduction | 124 (0.39%) | ko04744 | Organismal Systems |
| Glycerolipid metabolism | 122 (0.39%) | ko00561 | Metabolism |
| Glutathione metabolism | 120 (0.38%) | ko00480 | Metabolism |
| Cocaine addiction | 114 (0.36%) | ko05030 | Human Diseases |
| Sphingolipid metabolism | 114 (0.36%) | ko00600 | Metabolism |
| DNA replication | 110 (0.35%) | ko03030 | Genetic Information Processing |
| Tryptophan metabolism | 109 (0.35%) | ko00380 | Metabolism |
| Proteasome | 109 (0.35%) | ko03050 | Genetic Information Processing |
| Other types of O-glycan biosynthesis | 108 (0.34%) | ko00514 | Metabolism |
| Glycine, serine and threonine metabolism | 107 (0.34%) | ko00260 | Metabolism |
| Ether lipid metabolism | 107 (0.34%) | ko00565 | Metabolism |
| Tyrosine metabolism | 106 (0.34%) | ko00350 | Metabolism |
| Linoleic acid metabolism | 105 (0.33%) | ko00591 | Metabolism |
| Base excision repair | 102 (0.32%) | ko03410 | Genetic Information Processing |
| Malaria | 97 (0.31%) | ko05144 | Human Diseases |
| beta-Alanine metabolism | 96 (0.31%) | ko00410 | Metabolism |
| Pentose phosphate pathway | 94 (0.3%) | ko00030 | Metabolism |
| African trypanosomiasis | 92 (0.29%) | ko05143 | Human Diseases |
| Nicotinate and nicotinamide metabolism | 92 (0.29%) | ko00760 | Metabolism |
| Alanine, aspartate and glutamate metabolism | 91 (0.29%) | ko00250 | Metabolism |
| Glycosaminoglycan biosynthesis - heparan sulfate | 89 (0.28%) | ko00534 | Metabolism |
| Homologous recombination | 88 (0.28%) | ko03440 | Genetic Information Processing |
| Glyoxylate and dicarboxylate metabolism | 86 (0.27%) | ko00630 | Metabolism |
| Circadian rhythm - mammal | 84 (0.27%) | ko04710 | Organismal Systems |
| Porphyrin and chlorophyll metabolism | 82 (0.26%) | ko00860 | Metabolism |
| Butanoate metabolism | 82 (0.26%) | ko00650 | Metabolism |
| Autoimmune thyroid disease | 81 (0.26%) | ko05320 | Human Diseases |
| MAPK signaling pathway - fly | 78 (0.25%) | ko04013 | Environmental Information Processing |
| Histidine metabolism | 78 (0.25%) | ko00340 | Metabolism |
| Allograft rejection | 77 (0.25%) | ko05330 | Human Diseases |
| SNARE interactions in vesicular transport | 76 (0.24%) | ko04130 | Genetic Information Processing |
| Fat digestion and absorption | 75 (0.24%) | ko04975 | Organismal Systems |
| Mismatch repair | 74 (0.24%) | ko03430 | Genetic Information Processing |
| Vitamin digestion and absorption | 73 (0.23%) | ko04977 | Organismal Systems |
| Collecting duct acid secretion | 73 (0.23%) | ko04966 | Organismal Systems |
| Glycosylphosphatidylinositol(GPI)-anchor biosynthesis | 73 (0.23%) | ko00563 | Metabolism |
| Protein export | 72 (0.23%) | ko03060 | Genetic Information Processing |
| Graft-versus-host disease | 69 (0.22%) | ko05332 | Human Diseases |
| Type I diabetes mellitus | 69 (0.22%) | ko04940 | Human Diseases |
| Terpenoid backbone biosynthesis | 66 (0.21%) | ko00900 | Metabolism |
| Biosynthesis of unsaturated fatty acids | 65 (0.21%) | ko01040 | Metabolism |
| Regulation of autophagy | 63 (0.2%) | ko04140 | Cellular Processes |
| Fatty acid elongation | 63 (0.2%) | ko00062 | Metabolism |
| Mucin type O-Glycan biosynthesis | 58 (0.18%) | ko00512 | Metabolism |
| Proximal tubule bicarbonate reclamation | 58 (0.18%) | ko04964 | Organismal Systems |
| Other glycan degradation | 57 (0.18%) | ko00511 | Metabolism |
| Glycosaminoglycan degradation | 55 (0.18%) | ko00531 | Metabolism |
| Phenylalanine metabolism | 54 (0.17%) | ko00360 | Metabolism |
| Intestinal immune network for IgA production | 54 (0.17%) | ko04672 | Organismal Systems |
| Glycosphingolipid biosynthesis - lacto and neolacto series | 52 (0.17%) | ko00601 | Metabolism |
| Selenocompound metabolism | 49 (0.16%) | ko00450 | Metabolism |
| Ubiquinone and other terpenoid-quinone biosynthesis | 46 (0.15%) | ko00130 | Metabolism |
| Non-homologous end-joining | 46 (0.15%) | ko03450 | Genetic Information Processing |
| Steroid biosynthesis | 45 (0.14%) | ko00100 | Metabolism |
| Glycosphingolipid biosynthesis - ganglio series | 44 (0.14%) | ko00604 | Metabolism |
| Pantothenate and CoA biosynthesis | 43 (0.14%) | ko00770 | Metabolism |
| Circadian rhythm - fly | 42 (0.13%) | ko04711 | Organismal Systems |
| alpha-Linolenic acid metabolism | 40 (0.13%) | ko00592 | Metabolism |
| Butirosin and neomycin biosynthesis | 39 (0.12%) | ko00524 | Metabolism |
| Glycosaminoglycan biosynthesis - keratan sulfate | 38 (0.12%) | ko00533 | Metabolism |
| Sulfur relay system | 38 (0.12%) | ko04122 | Genetic Information Processing |
| One carbon pool by folate | 37 (0.12%) | ko00670 | Metabolism |
| Glycosaminoglycan biosynthesis - chondroitin sulfate | 35 (0.11%) | ko00532 | Metabolism |
| Riboflavin metabolism | 34 (0.11%) | ko00740 | Metabolism |
| Glycosphingolipid biosynthesis - globo series | 34 (0.11%) | ko00603 | Metabolism |
| Caffeine metabolism | 32 (0.1%) | ko00232 | Metabolism |
| Asthma | 32 (0.1%) | ko05310 | Human Diseases |
| Renin-angiotensin system | 31 (0.1%) | ko04614 | Organismal Systems |
| Folate biosynthesis | 29 (0.09%) | ko00790 | Metabolism |
| Sulfur metabolism | 29 (0.09%) | ko00920 | Metabolism |
| Fatty acid biosynthesis | 24 (0.08%) | ko00061 | Metabolism |
| Synthesis and degradation of ketone bodies | 23 (0.07%) | ko00072 | Metabolism |
| Cyanoamino acid metabolism | 17 (0.05%) | ko00460 | Metabolism |
| Taurine and hypotaurine metabolism | 16 (0.05%) | ko00430 | Metabolism |
| Vitamin B6 metabolism | 14 (0.04%) | ko00750 | Metabolism |
| Nicotine addiction | 13 (0.04%) | ko05033 | Human Diseases |
| Phenylalanine, tyrosine and tryptophan biosynthesis | 13 (0.04%) | ko00400 | Metabolism |
| D-Arginine and D-ornithine metabolism | 11 (0.04%) | ko00472 | Metabolism |
| Valine, leucine and isoleucine biosynthesis | 11 (0.04%) | ko00290 | Metabolism |
| Thiamine metabolism | 10 (0.03%) | ko00730 | Metabolism |
| Lipoic acid metabolism | 6 (0.02%) | ko00785 | Metabolism |
| Biotin metabolism | 6 (0.02%) | ko00780 | Metabolism |
| D-Glutamine and D-glutamate metabolism | 5 (0.02%) | ko00471 | Metabolism |
| Insect hormone biosynthesis | 4 (0.01%) | ko00981 | Metabolism |
| Polyketide sugar unit biosynthesis | 2 (0.01%) | ko00523 | Metabolism |
| Lysine biosynthesis | 2 (0.01%) | ko00300 | Metabolism |

**Table S3 Enrichment of KEGG pathway in the differentially expressed unigenes between S and B populations.**

| **Pathway** | **DEGs with pathway annotation (1717)** | **All genes with pathway annotation (31405)** | **Pvalue** | **Qvalue** | **Pathway ID** |
| --- | --- | --- | --- | --- | --- |
| [Ribosome](file:///C:\Users\shulongf\Dropbox\Papers\As%20author\2014%20RNA%20seq\SI_DGEs%20S_B.xlsx#RANGE!gene1) | 87 (5.07%) | 254 (0.81%) | 0.000 | 0.000 | ko03010 |
| [Oxidative phosphorylation](file:///C:\Users\shulongf\Dropbox\Papers\As%20author\2014%20RNA%20seq\SI_DGEs%20S_B.xlsx#RANGE!gene2) | 82 (4.78%) | 311 (0.99%) | 0.000 | 0.000 | ko00190 |
| [Parkinson's disease](file:///C:\Users\shulongf\Dropbox\Papers\As%20author\2014%20RNA%20seq\SI_DGEs%20S_B.xlsx#RANGE!gene3) | 74 (4.31%) | 310 (0.99%) | 0.000 | 0.000 | ko05012 |
| [Proteasome](file:///C:\Users\shulongf\Dropbox\Papers\As%20author\2014%20RNA%20seq\SI_DGEs%20S_B.xlsx#RANGE!gene4) | 44 (2.56%) | 109 (0.35%) | 0.000 | 0.000 | ko03050 |
| [Metabolic pathways](file:///C:\Users\shulongf\Dropbox\Papers\As%20author\2014%20RNA%20seq\SI_DGEs%20S_B.xlsx#RANGE!gene5) | 313 (18.23%) | 3796 (12.09%) | 0.000 | 0.000 | ko01100 |
| [Alzheimer's disease](file:///C:\Users\shulongf\Dropbox\Papers\As%20author\2014%20RNA%20seq\SI_DGEs%20S_B.xlsx#RANGE!gene6) | 88 (5.13%) | 753 (2.4%) | 0.000 | 0.000 | ko05010 |
| [Lysosome](file:///C:\Users\shulongf\Dropbox\Papers\As%20author\2014%20RNA%20seq\SI_DGEs%20S_B.xlsx#RANGE!gene7) | 66 (3.84%) | 493 (1.57%) | 0.000 | 0.000 | ko04142 |
| [Phototransduction - fly](file:///C:\Users\shulongf\Dropbox\Papers\As%20author\2014%20RNA%20seq\SI_DGEs%20S_B.xlsx#RANGE!gene8) | 28 (1.63%) | 125 (0.4%) | 0.000 | 0.000 | ko04745 |
| [Phagosome](file:///C:\Users\shulongf\Dropbox\Papers\As%20author\2014%20RNA%20seq\SI_DGEs%20S_B.xlsx#RANGE!gene9) | 70 (4.08%) | 575 (1.83%) | 0.000 | 0.000 | ko04145 |
| [Vibrio cholerae infection](file:///C:\Users\shulongf\Dropbox\Papers\As%20author\2014%20RNA%20seq\SI_DGEs%20S_B.xlsx#RANGE!gene10) | 57 (3.32%) | 438 (1.39%) | 0.000 | 0.000 | ko05110 |
| [Antigen processing and presentation](file:///C:\Users\shulongf\Dropbox\Papers\As%20author\2014%20RNA%20seq\SI_DGEs%20S_B.xlsx#RANGE!gene11) | 34 (1.98%) | 194 (0.62%) | 0.000 | 0.000 | ko04612 |
| [Pathogenic Escherichia coli infection](file:///C:\Users\shulongf\Dropbox\Papers\As%20author\2014%20RNA%20seq\SI_DGEs%20S_B.xlsx#RANGE!gene12) | 56 (3.26%) | 458 (1.46%) | 0.000 | 0.000 | ko05130 |
| [Glycolysis / Gluconeogenesis](file:///C:\Users\shulongf\Dropbox\Papers\As%20author\2014%20RNA%20seq\SI_DGEs%20S_B.xlsx#RANGE!gene13) | 34 (1.98%) | 229 (0.73%) | 0.000 | 0.000 | ko00010 |
| [Citrate cycle (TCA cycle)](file:///C:\Users\shulongf\Dropbox\Papers\As%20author\2014%20RNA%20seq\SI_DGEs%20S_B.xlsx#RANGE!gene14) | 24 (1.4%) | 134 (0.43%) | 0.000 | 0.000 | ko00020 |
| [Protein processing in endoplasmic reticulum](file:///C:\Users\shulongf\Dropbox\Papers\As%20author\2014%20RNA%20seq\SI_DGEs%20S_B.xlsx#RANGE!gene15) | 87 (5.07%) | 950 (3.02%) | 0.000 | 0.000 | ko04141 |
| [Protein export](file:///C:\Users\shulongf\Dropbox\Papers\As%20author\2014%20RNA%20seq\SI_DGEs%20S_B.xlsx#RANGE!gene16) | 15 (0.87%) | 72 (0.23%) | 0.000 | 0.000 | ko03060 |
| [Gastric acid secretion](file:///C:\Users\shulongf\Dropbox\Papers\As%20author\2014%20RNA%20seq\SI_DGEs%20S_B.xlsx#RANGE!gene17) | 36 (2.1%) | 315 (1%) | 0.000 | 0.000 | ko04971 |
| [Collecting duct acid secretion](file:///C:\Users\shulongf\Dropbox\Papers\As%20author\2014%20RNA%20seq\SI_DGEs%20S_B.xlsx#RANGE!gene18) | 14 (0.82%) | 73 (0.23%) | 0.000 | 0.000 | ko04966 |
| [Amphetamine addiction](file:///C:\Users\shulongf\Dropbox\Papers\As%20author\2014%20RNA%20seq\SI_DGEs%20S_B.xlsx#RANGE!gene19) | 26 (1.51%) | 201 (0.64%) | 0.000 | 0.001 | ko05031 |
| [Alcoholism](file:///C:\Users\shulongf\Dropbox\Papers\As%20author\2014%20RNA%20seq\SI_DGEs%20S_B.xlsx#RANGE!gene20) | 40 (2.33%) | 380 (1.21%) | 0.000 | 0.001 | ko05034 |
| [N-Glycan biosynthesis](file:///C:\Users\shulongf\Dropbox\Papers\As%20author\2014%20RNA%20seq\SI_DGEs%20S_B.xlsx#RANGE!gene21) | 22 (1.28%) | 163 (0.52%) | 0.000 | 0.001 | ko00510 |
| [Spliceosome](file:///C:\Users\shulongf\Dropbox\Papers\As%20author\2014%20RNA%20seq\SI_DGEs%20S_B.xlsx#RANGE!gene22) | 76 (4.43%) | 910 (2.9%) | 0.000 | 0.002 | ko03040 |
| [Pentose phosphate pathway](file:///C:\Users\shulongf\Dropbox\Papers\As%20author\2014%20RNA%20seq\SI_DGEs%20S_B.xlsx#RANGE!gene23) | 15 (0.87%) | 94 (0.3%) | 0.000 | 0.002 | ko00030 |
| [Pyruvate metabolism](file:///C:\Users\shulongf\Dropbox\Papers\As%20author\2014%20RNA%20seq\SI_DGEs%20S_B.xlsx#RANGE!gene24) | 20 (1.16%) | 149 (0.47%) | 0.000 | 0.002 | ko00620 |
| [Oocyte meiosis](file:///C:\Users\shulongf\Dropbox\Papers\As%20author\2014%20RNA%20seq\SI_DGEs%20S_B.xlsx#RANGE!gene25) | 38 (2.21%) | 381 (1.21%) | 0.000 | 0.003 | ko04114 |
| [Rheumatoid arthritis](file:///C:\Users\shulongf\Dropbox\Papers\As%20author\2014%20RNA%20seq\SI_DGEs%20S_B.xlsx#RANGE!gene26) | 20 (1.16%) | 154 (0.49%) | 0.000 | 0.003 | ko05323 |
| [Systemic lupus erythematosus](file:///C:\Users\shulongf\Dropbox\Papers\As%20author\2014%20RNA%20seq\SI_DGEs%20S_B.xlsx#RANGE!gene27) | 19 (1.11%) | 143 (0.46%) | 0.000 | 0.003 | ko05322 |
| [Huntington's disease](file:///C:\Users\shulongf\Dropbox\Papers\As%20author\2014%20RNA%20seq\SI_DGEs%20S_B.xlsx#RANGE!gene28) | 118 (6.87%) | 1612 (5.13%) | 0.001 | 0.007 | ko05016 |
| [Aminoacyl-tRNA biosynthesis](file:///C:\Users\shulongf\Dropbox\Papers\As%20author\2014%20RNA%20seq\SI_DGEs%20S_B.xlsx#RANGE!gene29) | 19 (1.11%) | 154 (0.49%) | 0.001 | 0.007 | ko00970 |
| [Other types of O-glycan biosynthesis](file:///C:\Users\shulongf\Dropbox\Papers\As%20author\2014%20RNA%20seq\SI_DGEs%20S_B.xlsx#RANGE!gene30) | 15 (0.87%) | 108 (0.34%) | 0.001 | 0.007 | ko00514 |
| [Synaptic vesicle cycle](file:///C:\Users\shulongf\Dropbox\Papers\As%20author\2014%20RNA%20seq\SI_DGEs%20S_B.xlsx#RANGE!gene31) | 23 (1.34%) | 205 (0.65%) | 0.001 | 0.007 | ko04721 |
| [Galactose metabolism](file:///C:\Users\shulongf\Dropbox\Papers\As%20author\2014%20RNA%20seq\SI_DGEs%20S_B.xlsx#RANGE!gene32) | 19 (1.11%) | 163 (0.52%) | 0.002 | 0.012 | ko00052 |
| [PPAR signaling pathway](file:///C:\Users\shulongf\Dropbox\Papers\As%20author\2014%20RNA%20seq\SI_DGEs%20S_B.xlsx#RANGE!gene33) | 24 (1.4%) | 230 (0.73%) | 0.002 | 0.014 | ko03320 |
| [Gap junction](file:///C:\Users\shulongf\Dropbox\Papers\As%20author\2014%20RNA%20seq\SI_DGEs%20S_B.xlsx#RANGE!gene34) | 28 (1.63%) | 284 (0.9%) | 0.002 | 0.014 | ko04540 |
| [Glutathione metabolism](file:///C:\Users\shulongf\Dropbox\Papers\As%20author\2014%20RNA%20seq\SI_DGEs%20S_B.xlsx#RANGE!gene35) | 15 (0.87%) | 120 (0.38%) | 0.002 | 0.016 | ko00480 |
| [Propanoate metabolism](file:///C:\Users\shulongf\Dropbox\Papers\As%20author\2014%20RNA%20seq\SI_DGEs%20S_B.xlsx#RANGE!gene36) | 16 (0.93%) | 132 (0.42%) | 0.002 | 0.016 | ko00640 |
| [Fat digestion and absorption](file:///C:\Users\shulongf\Dropbox\Papers\As%20author\2014%20RNA%20seq\SI_DGEs%20S_B.xlsx#RANGE!gene37) | 11 (0.64%) | 75 (0.24%) | 0.002 | 0.016 | ko04975 |
| [Cardiac muscle contraction](file:///C:\Users\shulongf\Dropbox\Papers\As%20author\2014%20RNA%20seq\SI_DGEs%20S_B.xlsx#RANGE!gene38) | 36 (2.1%) | 400 (1.27%) | 0.002 | 0.016 | ko04260 |
| [Insulin signaling pathway](file:///C:\Users\shulongf\Dropbox\Papers\As%20author\2014%20RNA%20seq\SI_DGEs%20S_B.xlsx#RANGE!gene39) | 52 (3.03%) | 634 (2.02%) | 0.002 | 0.016 | ko04910 |
| [Phototransduction](file:///C:\Users\shulongf\Dropbox\Papers\As%20author\2014%20RNA%20seq\SI_DGEs%20S_B.xlsx#RANGE!gene40) | 15 (0.87%) | 124 (0.39%) | 0.003 | 0.020 | ko04744 |
| [Vasopressin-regulated water reabsorption](file:///C:\Users\shulongf\Dropbox\Papers\As%20author\2014%20RNA%20seq\SI_DGEs%20S_B.xlsx#RANGE!gene41) | 29 (1.69%) | 310 (0.99%) | 0.004 | 0.022 | ko04962 |
| [Tryptophan metabolism](file:///C:\Users\shulongf\Dropbox\Papers\As%20author\2014%20RNA%20seq\SI_DGEs%20S_B.xlsx#RANGE!gene42) | 13 (0.76%) | 109 (0.35%) | 0.007 | 0.039 | ko00380 |
| [Long-term potentiation](file:///C:\Users\shulongf\Dropbox\Papers\As%20author\2014%20RNA%20seq\SI_DGEs%20S_B.xlsx#RANGE!gene43) | 31 (1.81%) | 353 (1.12%) | 0.007 | 0.039 | ko04720 |
| [Arginine and proline metabolism](file:///C:\Users\shulongf\Dropbox\Papers\As%20author\2014%20RNA%20seq\SI_DGEs%20S_B.xlsx#RANGE!gene44) | 17 (0.99%) | 160 (0.51%) | 0.007 | 0.039 | ko00330 |
| [Legionellosis](file:///C:\Users\shulongf\Dropbox\Papers\As%20author\2014%20RNA%20seq\SI_DGEs%20S_B.xlsx#RANGE!gene45) | 20 (1.16%) | 203 (0.65%) | 0.008 | 0.045 | ko05134 |
| [Valine, leucine and isoleucine degradation](file:///C:\Users\shulongf\Dropbox\Papers\As%20author\2014%20RNA%20seq\SI_DGEs%20S_B.xlsx#RANGE!gene46) | 18 (1.05%) | 177 (0.56%) | 0.008 | 0.046 | ko00280 |
| [Pancreatic secretion](file:///C:\Users\shulongf\Dropbox\Papers\As%20author\2014%20RNA%20seq\SI_DGEs%20S_B.xlsx#RANGE!gene47) | 24 (1.4%) | 260 (0.83%) | 0.009 | 0.047 | ko04972 |
| [Glycosaminoglycan biosynthesis - heparan sulfate](file:///C:\Users\shulongf\Dropbox\Papers\As%20author\2014%20RNA%20seq\SI_DGEs%20S_B.xlsx#RANGE!gene48) | 11 (0.64%) | 89 (0.28%) | 0.009 | 0.048 | ko00534 |
| [Starch and sucrose metabolism](file:///C:\Users\shulongf\Dropbox\Papers\As%20author\2014%20RNA%20seq\SI_DGEs%20S_B.xlsx#RANGE!gene49) | 27 (1.57%) | 304 (0.97%) | 0.009 | 0.048 | ko00500 |

**Table S4 Enrichment of KEGG pathway in the differentially expressed unigenes between S and T populations.**

| **Pathway** | **DEGs with pathway annotation (1529)** | **All genes with pathway annotation (31405)** | **Pvalue** | **Qvalue** | **Pathway ID** |
| --- | --- | --- | --- | --- | --- |
| [Ribosome](file:///C:\Users\shulongf\Dropbox\Papers\As%20author\2014%20RNA%20seq\SI_DGEs%20S_T.xlsx#RANGE!gene1) | 87 (5.69%) | 254 (0.81%) | 0.000 | 0.000 | ko03010 |
| [Oxidative phosphorylation](file:///C:\Users\shulongf\Dropbox\Papers\As%20author\2014%20RNA%20seq\SI_DGEs%20S_T.xlsx#RANGE!gene2) | 73 (4.77%) | 311 (0.99%) | 0.000 | 0.000 | ko00190 |
| [Parkinson's disease](file:///C:\Users\shulongf\Dropbox\Papers\As%20author\2014%20RNA%20seq\SI_DGEs%20S_T.xlsx#RANGE!gene3) | 68 (4.45%) | 310 (0.99%) | 0.000 | 0.000 | ko05012 |
| [Proteasome](file:///C:\Users\shulongf\Dropbox\Papers\As%20author\2014%20RNA%20seq\SI_DGEs%20S_T.xlsx#RANGE!gene4) | 41 (2.68%) | 109 (0.35%) | 0.000 | 0.000 | ko03050 |
| [Alzheimer's disease](file:///C:\Users\shulongf\Dropbox\Papers\As%20author\2014%20RNA%20seq\SI_DGEs%20S_T.xlsx#RANGE!gene5) | 82 (5.36%) | 753 (2.4%) | 0.000 | 0.000 | ko05010 |
| [Antigen processing and presentation](file:///C:\Users\shulongf\Dropbox\Papers\As%20author\2014%20RNA%20seq\SI_DGEs%20S_T.xlsx#RANGE!gene6) | 35 (2.29%) | 194 (0.62%) | 0.000 | 0.000 | ko04612 |
| [Lysosome](file:///C:\Users\shulongf\Dropbox\Papers\As%20author\2014%20RNA%20seq\SI_DGEs%20S_T.xlsx#RANGE!gene7) | 60 (3.92%) | 493 (1.57%) | 0.000 | 0.000 | ko04142 |
| [Pathogenic Escherichia coli infection](file:///C:\Users\shulongf\Dropbox\Papers\As%20author\2014%20RNA%20seq\SI_DGEs%20S_T.xlsx#RANGE!gene8) | 54 (3.53%) | 458 (1.46%) | 0.000 | 0.000 | ko05130 |
| [Vibrio cholerae infection](file:///C:\Users\shulongf\Dropbox\Papers\As%20author\2014%20RNA%20seq\SI_DGEs%20S_T.xlsx#RANGE!gene9) | 52 (3.4%) | 438 (1.39%) | 0.000 | 0.000 | ko05110 |
| [Metabolic pathways](file:///C:\Users\shulongf\Dropbox\Papers\As%20author\2014%20RNA%20seq\SI_DGEs%20S_T.xlsx#RANGE!gene10) | 260 (17%) | 3796 (12.09%) | 0.000 | 0.000 | ko01100 |
| [Phagosome](file:///C:\Users\shulongf\Dropbox\Papers\As%20author\2014%20RNA%20seq\SI_DGEs%20S_T.xlsx#RANGE!gene11) | 61 (3.99%) | 575 (1.83%) | 0.000 | 0.000 | ko04145 |
| [Phototransduction - fly](file:///C:\Users\shulongf\Dropbox\Papers\As%20author\2014%20RNA%20seq\SI_DGEs%20S_T.xlsx#RANGE!gene12) | 21 (1.37%) | 125 (0.4%) | 0.000 | 0.000 | ko04745 |
| [Glycolysis / Gluconeogenesis](file:///C:\Users\shulongf\Dropbox\Papers\As%20author\2014%20RNA%20seq\SI_DGEs%20S_T.xlsx#RANGE!gene13) | 27 (1.77%) | 229 (0.73%) | 0.000 | 0.000 | ko00010 |
| [Citrate cycle (TCA cycle)](file:///C:\Users\shulongf\Dropbox\Papers\As%20author\2014%20RNA%20seq\SI_DGEs%20S_T.xlsx#RANGE!gene14) | 19 (1.24%) | 134 (0.43%) | 0.000 | 0.000 | ko00020 |
| [Protein processing in endoplasmic reticulum](file:///C:\Users\shulongf\Dropbox\Papers\As%20author\2014%20RNA%20seq\SI_DGEs%20S_T.xlsx#RANGE!gene15) | 75 (4.91%) | 950 (3.02%) | 0.000 | 0.000 | ko04141 |
| [Protein export](file:///C:\Users\shulongf\Dropbox\Papers\As%20author\2014%20RNA%20seq\SI_DGEs%20S_T.xlsx#RANGE!gene16) | 13 (0.85%) | 72 (0.23%) | 0.000 | 0.001 | ko03060 |
| [Collecting duct acid secretion](file:///C:\Users\shulongf\Dropbox\Papers\As%20author\2014%20RNA%20seq\SI_DGEs%20S_T.xlsx#RANGE!gene17) | 13 (0.85%) | 73 (0.23%) | 0.000 | 0.001 | ko04966 |
| [Huntington's disease](file:///C:\Users\shulongf\Dropbox\Papers\As%20author\2014%20RNA%20seq\SI_DGEs%20S_T.xlsx#RANGE!gene18) | 110 (7.19%) | 1612 (5.13%) | 0.000 | 0.003 | ko05016 |
| [Glutathione metabolism](file:///C:\Users\shulongf\Dropbox\Papers\As%20author\2014%20RNA%20seq\SI_DGEs%20S_T.xlsx#RANGE!gene19) | 16 (1.05%) | 120 (0.38%) | 0.000 | 0.003 | ko00480 |
| [Alcoholism](file:///C:\Users\shulongf\Dropbox\Papers\As%20author\2014%20RNA%20seq\SI_DGEs%20S_T.xlsx#RANGE!gene20) | 35 (2.29%) | 380 (1.21%) | 0.000 | 0.003 | ko05034 |
| [Fat digestion and absorption](file:///C:\Users\shulongf\Dropbox\Papers\As%20author\2014%20RNA%20seq\SI_DGEs%20S_T.xlsx#RANGE!gene21) | 12 (0.78%) | 75 (0.24%) | 0.000 | 0.003 | ko04975 |
| [Amphetamine addiction](file:///C:\Users\shulongf\Dropbox\Papers\As%20author\2014%20RNA%20seq\SI_DGEs%20S_T.xlsx#RANGE!gene22) | 22 (1.44%) | 201 (0.64%) | 0.000 | 0.004 | ko05031 |
| [N-Glycan biosynthesis](file:///C:\Users\shulongf\Dropbox\Papers\As%20author\2014%20RNA%20seq\SI_DGEs%20S_T.xlsx#RANGE!gene23) | 18 (1.18%) | 163 (0.52%) | 0.001 | 0.011 | ko00510 |
| [Cardiac muscle contraction](file:///C:\Users\shulongf\Dropbox\Papers\As%20author\2014%20RNA%20seq\SI_DGEs%20S_T.xlsx#RANGE!gene24) | 34 (2.22%) | 400 (1.27%) | 0.001 | 0.013 | ko04260 |
| [Spliceosome](file:///C:\Users\shulongf\Dropbox\Papers\As%20author\2014%20RNA%20seq\SI_DGEs%20S_T.xlsx#RANGE!gene25) | 65 (4.25%) | 910 (2.9%) | 0.001 | 0.014 | ko03040 |
| [Proximal tubule bicarbonate reclamation](file:///C:\Users\shulongf\Dropbox\Papers\As%20author\2014%20RNA%20seq\SI_DGEs%20S_T.xlsx#RANGE!gene26) | 9 (0.59%) | 58 (0.18%) | 0.002 | 0.018 | ko04964 |
| [Arginine and proline metabolism](file:///C:\Users\shulongf\Dropbox\Papers\As%20author\2014%20RNA%20seq\SI_DGEs%20S_T.xlsx#RANGE!gene27) | 17 (1.11%) | 160 (0.51%) | 0.002 | 0.019 | ko00330 |
| [Pyruvate metabolism](file:///C:\Users\shulongf\Dropbox\Papers\As%20author\2014%20RNA%20seq\SI_DGEs%20S_T.xlsx#RANGE!gene28) | 16 (1.05%) | 149 (0.47%) | 0.003 | 0.022 | ko00620 |
| [Phototransduction](file:///C:\Users\shulongf\Dropbox\Papers\As%20author\2014%20RNA%20seq\SI_DGEs%20S_T.xlsx#RANGE!gene29) | 14 (0.92%) | 124 (0.39%) | 0.003 | 0.025 | ko04744 |
| [Gastric acid secretion](file:///C:\Users\shulongf\Dropbox\Papers\As%20author\2014%20RNA%20seq\SI_DGEs%20S_T.xlsx#RANGE!gene30) | 27 (1.77%) | 315 (1%) | 0.003 | 0.027 | ko04971 |
| [Rheumatoid arthritis](file:///C:\Users\shulongf\Dropbox\Papers\As%20author\2014%20RNA%20seq\SI_DGEs%20S_T.xlsx#RANGE!gene31) | 16 (1.05%) | 154 (0.49%) | 0.004 | 0.028 | ko05323 |
| [Glycosaminoglycan biosynthesis - heparan sulfate](file:///C:\Users\shulongf\Dropbox\Papers\As%20author\2014%20RNA%20seq\SI_DGEs%20S_T.xlsx#RANGE!gene32) | 11 (0.72%) | 89 (0.28%) | 0.004 | 0.030 | ko00534 |
| [Systemic lupus erythematosus](file:///C:\Users\shulongf\Dropbox\Papers\As%20author\2014%20RNA%20seq\SI_DGEs%20S_T.xlsx#RANGE!gene33) | 15 (0.98%) | 143 (0.46%) | 0.004 | 0.032 | ko05322 |
| [Insulin signaling pathway](file:///C:\Users\shulongf\Dropbox\Papers\As%20author\2014%20RNA%20seq\SI_DGEs%20S_T.xlsx#RANGE!gene34) | 46 (3.01%) | 634 (2.02%) | 0.005 | 0.035 | ko04910 |
| [Legionellosis](file:///C:\Users\shulongf\Dropbox\Papers\As%20author\2014%20RNA%20seq\SI_DGEs%20S_T.xlsx#RANGE!gene35) | 19 (1.24%) | 203 (0.65%) | 0.005 | 0.035 | ko05134 |
| [Synaptic vesicle cycle](file:///C:\Users\shulongf\Dropbox\Papers\As%20author\2014%20RNA%20seq\SI_DGEs%20S_T.xlsx#RANGE!gene36) | 19 (1.24%) | 205 (0.65%) | 0.006 | 0.038 | ko04721 |
| [Arachidonic acid metabolism](file:///C:\Users\shulongf\Dropbox\Papers\As%20author\2014%20RNA%20seq\SI_DGEs%20S_T.xlsx#RANGE!gene37) | 16 (1.05%) | 166 (0.53%) | 0.007 | 0.048 | ko00590 |

**Table S5 Enrichment of KEGG pathway in the differentially expressed unigenes between T and B populations.**

| **Pathway** | **DEGs with pathway annotation (615)** | **All genes with pathway annotation (31405)** | **Pvalue** | **Qvalue** | **Pathway ID** |
| --- | --- | --- | --- | --- | --- |
| [Morphine addiction](file:///C:\Users\shulongf\Dropbox\Papers\As%20author\2014%20RNA%20seq\SI_DGEs%20T_B.xlsx#RANGE!gene1) | 39 (6.34%) | 1081 (3.44%) | 0.000 | 0.030 | ko05032 |
| [Salivary secretion](file:///C:\Users\shulongf\Dropbox\Papers\As%20author\2014%20RNA%20seq\SI_DGEs%20T_B.xlsx#RANGE!gene2) | 17 (2.76%) | 327 (1.04%) | 0.000 | 0.030 | ko04970 |
